# La Grippe

**Published:** 1890-02

**Authors:** 


### "LA GRIPPE."

The sensational newspaper accounts of the ravages of influenza in European and Northern cities have unduly alarmed the populace and are proving quite a harvest to physicians throughout the country.

Simple cases of catarrhal fever diagnosed La Grippe either by the patients themselves or their physicians assume enormous proportions in the imaginations of the victims. Undoubted cases of influenza have, however, made their appearance, and new cases are developing on every hand.

The disease is usually ushered in by a chill or chilly sensations, pain in the back, headache, and the usual coryzal symptoms. Hyperpyrexia is a frequent concomitant, as is also marked prostration.

The affection readily yields to the usual remedies for catarrhal fever, though many cases prove quite obstinate, and a few deaths from this cause are reported.

On the whole the epidemic seems to be of rather a mild type, and not of unusual severity, as the laity have been led by the newspapers to believe. And while this undue alarm of the people is being used for gain by quacks and unscrupulous doctors with mercenary motives, it is of course a pleasure to the true physician to dispel the delusion and assure his patients of the harmless character of the disease.

---

THE *Medical Mirror*, edited by Dr. I. N. Love, of St. Louis, is a new journal which is destined to be popular with the profession. The journal like the editor is brimful of good fellowship.

THE *Southern Medical Record* comes to us much improved under its new management. With Dr. D. H. Howell, as business manager, and Drs. A. W. Griggs, W. P. Nicolson and F. O. Stockton, as editors the *Record* has a bright future before it.

THE *Dixie Doctor*, edited by Dr. T. H. Huzza, is a new medical journal published in Atlanta and does much credit to both editor and publishers.

